# Association of Parental Preconception Exposure to Phthalates and Phthalate Substitutes With Preterm Birth

**DOI:** 10.1001/jamanetworkopen.2020.2159

**Published:** 2020-04-07

**Authors:** Yu Zhang, Vicente Mustieles, Jennifer Yland, Joseph M. Braun, Paige L. Williams, Jill A. Attaman, Jennifer B. Ford, Antonia M. Calafat, Russ Hauser, Carmen Messerlian

**Affiliations:** 1 Department of Environmental Health, Harvard T.H. Chan School of Public Health, Boston, Massachusetts; 2 School of Health Humanities, Peking University Health Science Center, Beijing, China; 3 University of Granada, Center for Biomedical Research, Instituto de Investigación Biosanitaria Ibs Granada, Consortium for Biomedical Research in Epidemiology and Public Health (CIBERESP), Granada, Spain; 4 Department of Epidemiology, Harvard T.H. Chan School of Public Health, Boston, Massachusetts; 5 Department of Epidemiology, Brown University School of Public Health, Providence, Rhode Island; 6 Department of Biostatistics, Harvard T.H. Chan School of Public Health, Boston, Massachusetts; 7 Department of Obstetrics and Gynecology, Massachusetts General Hospital Fertility Center, Boston; 8 National Center for Environmental Health, Centers for Disease Control and Prevention, Atlanta, Georgia

## Abstract

**Importance:**

Although phthalate exposure during pregnancy has been associated with preterm birth, the association of preconception exposure in either parent with preterm birth constitutes a knowledge gap.

**Objective:**

To examine the association of paternal and maternal preconception urinary concentrations of biomarkers of phthalates and phthalate substitutes with singleton preterm birth.

**Design, Setting, and Participants:**

This study, conducted at an academic fertility center in Boston, Massachusetts, included a prospective preconception cohort of subfertile couples comprising 419 mothers and 229 fathers and their 420 live-born singleton offspring born between January 1, 2005, and December 31, 2018. Statistical analysis was performed from August 1 to October 31, 2019.

**Exposures:**

Urinary concentrations of metabolites of phthalates and phthalate substitutes obtained before conception.

**Main Outcomes and Measures:**

Gestational age was abstracted from delivery records and validated using the American College of Obstetricians and Gynecologists guidelines for births after medically assisted reproduction. The risk ratio (RR) of preterm birth (live birth before 37 completed weeks’ gestation) was estimated in association with urinary concentrations of 11 individual phthalate metabolites, the molar sum of 4 di-(2-ethylhexyl) phthalate (ΣDEHP) metabolites, and 2 metabolites of 1,2-cyclohexane dicarboxylic acid diisononyl ester (DINCH, a nonphthalate plasticizer substitute) using modified Poisson regression models adjusted for covariates.

**Results:**

The mean (SD) age of the 419 mothers was 34.7 (4.0) years, the mean (SD) age of the 229 fathers was 36.0 (4.5) years, and the mean (SD) gestational age of the 420 singleton children (217 boys) was 39.3 (1.7) weeks, with 34 (8%) born preterm. In adjusted models, maternal preconception ΣDEHP concentrations (RR, 1.50; 95% CI, 1.09-2.06; *P* = .01) and cyclohexane-1,2-dicarboxylic acid monohydroxy isononyl ester (MHiNCH, a metabolite of DINCH) concentrations (RR, 1.70; 95% CI, 0.89-3.24; *P* = .11) were associated with an increased risk of preterm birth. After additional adjustment for prenatal ΣDEHP or MHiNCH concentrations, the association of maternal preconception exposure to ΣDEHP and preterm birth remained robust (RR, 1.69; 95% CI, 1.17-2.44; *P* = .006), while the association of maternal preconception exposure to MHiNCH and preterm birth was attenuated (RR, 1.17; 95% CI, 0.49-2.81*; P* = .72). The remaining urinary metabolites examined in either parent showed no association with preterm birth.

**Conclusions and Relevance:**

In this prospective cohort of subfertile couples, maternal preconception exposure to ΣDEHP metabolites was associated with an increased risk of preterm birth. The results suggest that female exposure to select phthalate plasticizers during the preconception period may be a potential risk factor for adverse pregnancy outcomes, which may need to be considered in preconception care strategies.

## Key Points

**Question:**

Are paternal and maternal preconception urinary metabolite concentrations of phthalates and phthalate substitutes associated with singleton preterm birth?

**Findings:**

In this preconception cohort study of 420 singleton infants, maternal preconception urinary concentrations of di-(2-ethylhexyl) phthalate and di(isononyl)cyclohexane-1,2-dicarboxylate (a nonphthalate plasticizer substitute) were associated with an increased risk of preterm birth. Fathers’ preconception concentrations of these biomarkers were not associated with preterm birth in offspring.

**Meaning:**

Female exposure to select plasticizers during the preconception period may be a potential risk factor for adverse pregnancy outcomes.

## Teaser

This cohort study examines the association of paternal and maternal preconception urinary concentrations of biomarkers of phthalates and phthalate substitutes with singleton preterm birth.

## Introduction

Preterm birth is the factor most strongly associated with neonatal mortality and long-term morbidity globally.^1,2,3^ In the United States, 1 in 10 pregnancies is delivered preterm, accounting for approximately 380 000 births per year.^4^ Beyond the increased risk of early death, the long-term consequences among surviving infants may be associated with neurologic, respiratory, and gastrointestinal disorders as well as cardiometabolic disease during adulthood.^1,5,6,7,8^

Preterm birth is a complex and heterogeneous condition with multiple etiopathogenic processes triggering early parturition.^3,9^ Although some risk factors for preterm birth have been identified, including maternal age, race/ethnicity, socioeconomic status, smoking during pregnancy, infection, and multiple gestations, these factors account for less than half of all cases, and underlying mechanisms remain largely unknown.^1,3,10,11,12,13^ There is increasing evidence of an association between environmental exposures during pregnancy (including air pollution and chemicals such as phthalates) and preterm birth.^14,15,16,17,18,19,20^

Phthalates are a family of chemicals widely used in many consumer products. Phthalates are known reproductive and developmental toxicants in experimental animals^21,22^ and are suspected to produce similar effects in humans.^23,24,25^ Human exposure to phthalates is ubiquitous in the United States, Europe, and elsewhere.^26,27,28^ Regulation of some phthalates has prompted the use of plasticizer replacement chemicals such as 1,2-cyclohexane dicarboxylic acid diisononyl ester (DINCH). Substitution of phthalates with DINCH warrants further screening in human populations^29^ because its metabolites are biologically active and understudied.^30^

The maternal preconception period remains an important but largely unexplored critical window of exposure for perinatal and infant outcomes.^31^ Even less is known about the association of paternal preconception exposures with offspring health.^32^ Environmental-epigenetic mechanisms in the preconception and periconception period are likely associated with the etiopathologic characteristics of preterm birth.^33,34,35^ However, studies addressing the association of parental preconception exposure to phthalates with outcomes in gametes, fertilization, implantation, placentation, and gestation are limited.^31^ Therefore, we aimed to investigate whether higher paternal and maternal preconception urinary concentrations of metabolites of phthalates and phthalate substitutes were associated with an increased risk of preterm birth among couples undergoing fertility care.

## Methods

### Study Cohort

The Environment and Reproductive Health (EARTH) Study is an ongoing prospective preconception cohort of couples seeking fertility evaluation and medically assisted reproductive treatment at the Massachusetts General Hospital Fertility Center. The EARTH Study was designed to investigate environmental and nutritional factors for both women and men across preconception and prenatal periods in association with fertility, pregnancy, and birth outcomes. The cohort has been described elsewhere.^36^ In brief, women aged 18 to 46 years and men aged 18 to 55 years, using their own gametes, were eligible. Participants enrolled independently or as a couple and were followed up from study entry through their fertility care, pregnancy, and labor and delivery. The present analysis included 419 female and 229 male EARTH cohort participants who gave birth to a singleton infant between January 1, 2005, and December 31, 2018, for whom we had at least 1 urine sample quantified for biomarker metabolites during the period before conception of the index pregnancy. One singleton live birth was from a male participant enrolled without a female partner, thus leaving 228 couples (Figure). Trained staff explained the study details to participants and answered questions before obtaining written informed consent. The study was approved by the Massachusetts General Hospital, Harvard T.H. Chan School of Public Health, and the Centers for Disease Control and Prevention Institutional Review Boards. This study followed the Strengthening the Reporting of Observational Studies in Epidemiology (STROBE) reporting guideline.

**Figure.**
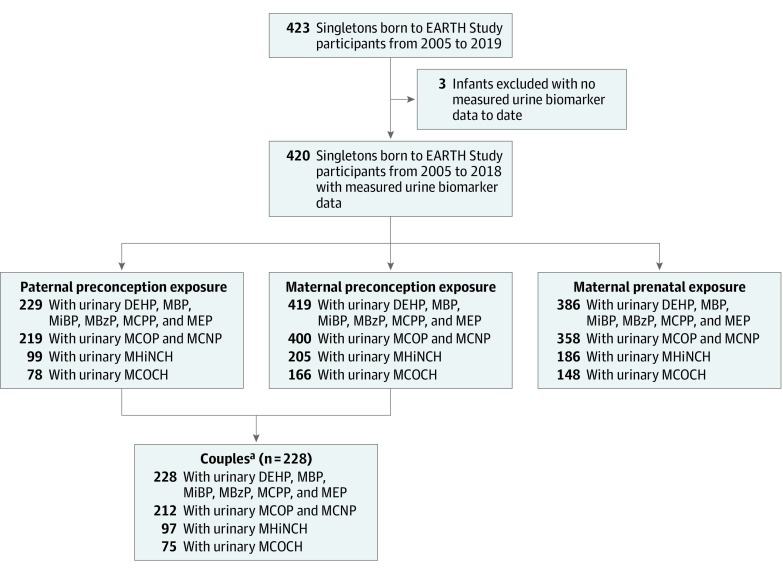
Participant Flowchart of Metabolites of Phthalates and Phthalate Substitutes Data Available in the Environment and Reproductive Health (EARTH) Study, 2005-2018. DEHP indicates di(2-ethylhexyl) phthalate; MBP, mono-n-butyl phthalate; MiBP, mono-isobutyl phthalate; MBzP, monobenzyl phthalate; MCPP, mono(3-carboxypropyl) phthalate; MCOP, monocarboxyisooctyl phthalate; MCNP, monocarboxyisononyl phthalate; MEP, monoethyl phthalate; MHiNCH, cyclohexane-1,2-dicarboxylic acid monohydroxy isononyl ester; and MCOCH, cyclohexane-1,2-dicarboxylic acid monocarboxyisooctyl ester. ^a^One male participant joined without a female partner, leaving 228 couples.

### Exposure Assessment

Male and female participants provided 1 spot urine sample at study entry. Women provided up to 2 additional spot urine samples per fertility treatment cycle: one obtained during the follicular phase of the cycle (days 3-9) and the other on the day of the fertility procedure. Men provided an additional spot urine sample per cycle on the day when their female partner underwent the fertility procedure. Women also provided 1 spot urine sample per trimester at a median of 6, 21, and 35 weeks’ gestation. We used the biomarker concentrations from the multiple urine samples obtained per participant from study entry up to and including the samples from the treatment cycle of conception of the index birth to estimate the mean exposure in the preconception window.

Urine samples were collected in polypropylene specimen cups, and the specific gravity (SG) of each sample was quantified with a handheld refractometer (National Instrument Company Inc). The urine samples were then divided into aliquots, frozen for long-term storage at −80 °C, and shipped on dry ice overnight to the Centers for Disease Control and Prevention (Atlanta, Georgia). For each urine sample, the concentrations of metabolites of phthalates and phthalate substitutes were quantified using solid-phase extraction coupled with high-performance liquid chromatography–isotope dilution tandem mass spectrometry.^37^ The concentrations of the following phthalate metabolites were measured: monoethyl phthalate, mono-n-butyl phthalate (MBP), mono-isobutyl phthalate (MiBP), monobenzyl phthalate (MBzP), mono(2-ethylhexyl) phthalate (MEHP), mono(2-ethyl-5-hydroxyhexyl) phthalate (MEHHP), mono(2-ethyl-5-oxohexyl) phthalate (MEOHP), mono(2-ethyl-5-carboxypentyl) phthalate (MECPP), mono(3-carboxypropyl) phthalate, monocarboxyisooctyl phthalate, and monocarboxyisononyl phthalate. The urinary concentrations of 2 DINCH metabolites, cyclohexane-1,2-dicarboxylic acid monohydroxy isononyl ester (MHiNCH) and cyclohexane-1,2-dicarboxylic acid monocarboxyisooctyl ester (MCOCH), were also measured in a subset of participants (Figure). The limits of detection (LOD) ranged from 0.1 to 1.2 ng/mL. Concentrations below the LOD were assigned the LOD divided by the square root of 2.^38^

We calculated the molar sum of 4 di(2-ethylhexyl) phthalate (DEHP) metabolites by dividing each metabolite concentration by its molecular weight and then summing: ΣDEHP = {[MEHP × (1/278.34)] + [MEHHP × (1/294.34)] + [MEOHP × (1/292.33)] + [MECPP × (1/308.33)]}. We multiplied the molar sum by the molecular weight of MECPP (308.33) to convert ΣDEHP to nanograms per milliliter. We also calculated a summary measure of phthalate metabolites with antiandrogenic properties (ie, MEHP, MEHHP, MEOHP, MECPP, MBP, MiBP, and MBzP), as previously described.^39^ The summary estimate (ΣAAPhthalates) was calculated by multiplying the SG-adjusted concentration of each of these 7 individual metabolites by their antiandrogenic potency and summing the weighted concentrations: ΣAAPhthalates = MBP + (0.24 × MiBP) + (0.26 × MBzP) + (0.61 × MEHP) + (0.61 × MEHHP) + (0.61 × MEOHP) + (0.61 × MECPP).^39,40^

### Outcome Assessment

We abstracted gestational age in days from delivery records and validated it using the American College of Obstetricians and Gynecologists guidelines for dating births after medically assisted reproduction.^41^ The fertility treatment setting permitted us to estimate gestational age with high accuracy using in vitro fertilization protocol dates, substantially reducing the number of misclassifications of preterm births due to inaccuracies in pregnancy dating.^42^ For in vitro fertilization pregnancies, the gestational age was estimated as (Outcome Date − Transfer Date + 14 Days + Cycle Day of Transfer).^41^ For intrauterine insemination and nonmedically assisted or naturally conceived pregnancies, we used the birth date minus the cycle start date or the last menstrual period date. Preterm birth was defined as any live birth less than 37 completed weeks’ gestation (<259 days). We corrected for 3 pregnancies for which the medical delivery record estimates (criterion standard) differed from the American College of Obstetricians and Gynecologists–based estimates by more than 6 days through additional delivery record verification.

### Covariates

Data on paternal and maternal age, educational level, race/ethnicity, and smoking status were obtained from self-reported questionnaires administered at enrollment. Research study staff measured the height and weight of the participants at baseline, and body mass index was calculated as weight in kilograms divided by height in meters squared. The treating infertility physician diagnosed the underlying cause of infertility using the Society for Assisted Reproductive Technology (ART) definitions.^43,44^ The type of medically assisted reproduction used in the conception cycle of the index birth was abstracted from the electronic medical records by trained study staff and dichotomized as ART procedures (all in vitro fertilization protocols, including intracytoplasmic sperm injection) vs non-ART protocols (all intrauterine insemination or ovarian stimulation protocols as well as nonmedically assisted or naturally conceived).

### Statistical Analysis

Statistical analysis was performed from August 1 to October 1, 2019. To account for urinary dilution, each biomarker concentration was multiplied by [(SGp − 1)/(SGi − 1)], where SGi is the SG of the participant’s sample and SGp is the mean SG for all male (mean, 1.016) or all female (mean, 1.015) participants included in the study.^45^ The SG-adjusted biomarker concentrations were natural log-transformed to standardize the distribution and reduce the effect of extreme values. We estimated the geometric mean paternal and maternal preconception biomarker concentrations by averaging each participant’s natural log concentration obtained from study entry (baseline) and at each treatment cycle up to and including the cycle of the index conception of the singleton offspring. We calculated descriptive statistics for biomarker concentrations and the percentage of values below the LOD, as well as Spearman correlation coefficients for each natural log concentration between couples (paternal vs maternal preconception and preconception vs prenatal windows, using the mean concentration across 3 trimesters).

We examined the clinical and demographic characteristics, reported as mean (SD) values or as numbers and percentages, of study participants in the total cohort. We fit modified Poisson regression models to evaluate the association of continuous urinary biomarker concentrations with dichotomous preterm birth outcomes.^46^ Modified Poisson models were fit by using a log-link function with a Poisson distribution to yield estimated risk ratios (RRs) and 95% CIs for preterm birth for every natural log unit increase in metabolite concentration. We fit a separate model for each of the 13 individual biomarkers of interest as well for the ΣDEHP and ΣAAPhthalates summary measures.

We selected covariates a priori as potential confounders based on substantive knowledge using a directed acyclic graph (eFigure in the Supplement) and examined unadjusted and covariate-adjusted results. All statistical models were adjusted for ART vs non-ART to control for mode of conception and indirectly for the underlying cause of infertility. Maternal preconception window covariate-adjusted models included maternal age and body mass index (continuous), maternal educational level (<college, college, or graduate degree), smoking status (never smoked or ever smoked, defined as a current or former smoker), and treatment type (ART or non-ART). Paternal preconception window covariate-adjusted models included paternal and maternal age and body mass index (continuous), paternal and maternal smoking (ever or never), maternal educational level (<college, college, or graduate degree), and treatment type (ART or non-ART). We further adjusted for partner’s preconception phthalate concentrations and maternal prenatal phthalate concentrations (averaged natural log concentrations across 3 trimesters), in additional covariate-adjusted models. All statistical analyses were performed with SAS, version 9.4 (SAS Institute Inc). Statistical tests were 2-tailed, and *P* < .05 was considered statistically significant.

### Sensitivity Analysis

First, we restricted the maternal preconception analyses to 228 couples to obtain more comparable results across models within couples. Second, to examine potential differences by infant sex, we stratified analyses and estimated sex-specific RRs and 95% CIs. Effect-measure modification *P* values were calculated for the interaction term (sex × urinary biomarker concentration). *P* < .20 was considered potential evidence of effect modification by infant sex on the multiplicative scale. Third, because our analyses on DINCH metabolites MHiNCH and MCOCH were limited by low detection rates, we dichotomized these parental preconception biomarkers by their median values and fit modified Poisson regression models for dichotomous DINCH metabolite concentrations and preterm birth, adjusting for covariates. We also fit multivariable general linear regression models for continuous parental preconception concentrations of MHiNCH and MCOCH with continuous gestational age to address concerns of lower power given the smaller sample size and therefore smaller number of cases of preterm birth in this subset in which DINCH biomarkers were measured. Coefficient estimates and 95% CIs represent the mean difference in gestational age for each natural log unit increase in urinary DINCH biomarker concentration. To assess the linearity assumption of our positive maternal DEHP results, we fit models across biomarker quartiles and estimated *P* values for trend across quartiles.

## Results

### Study Cohort

The study cohort included 419 mothers (mean [SD] age, 34.7 [4.0] years; mean [SD] body mass index, 24.1 [4.3]) and 229 fathers (mean [SD] age, 36.0 [4.5] years; mean [SD] body mass index, 27.7 [6.1]) (228 couples) at the time of enrollment (Table 1). Among the 420 singleton infants, the mean (SD) gestational age was 39.3 (1.7) weeks, with 34 infants (8%) born preterm (Table 2). The mean (SD) birth weight was 3363 (551) g, with 20 infants (5%) born with low birth weight (<2500 g).

**Table 1.** Parental Characteristics From Participants in the Environment and Reproductive Health Study, 2005-2018

Characteristic	Participants, No. (%)	
	Mothers (n = 419)	Fathers (n = 229)
Age, y		
Mean (SD)	34.7 (4.0)	36.0 (4.5)
>35	173 (41)	128 (56)
Race/ethnicity		
White	355 (85)	201 (88)
Black	11 (3)	4 (2)
Asian	36 (9)	15 (7)
Other	17 (4)	9 (4)
Body mass index^a^		
Mean (SD)	24.1 (4.3)	27.7 (6.1)
>25	132 (32)	158 (69)
Educational level		
<College	55 (13)	76 (33)
College graduate	137 (33)	64 (28)
Graduate degree	227 (54)	85 (37)
Smoking status		
Never	317 (76)	159 (69)
Ever (former or current)	102 (24)	70 (31)
Infertility diagnosis		
Male factor	101 (24)	70 (31)
Female factor	132 (32)	65 (28)
Unexplained	186 (44)	94 (41)
Primiparous	348 (83)	NA

Abbreviation: NA, not applicable.

^a^ Calculated as weight in kilograms divided by height in meters squared.

**Table 2.** Birth Characteristics of Singleton Infants From the Environment and Reproductive Health Study, 2005-2018

Characteristic	Singleton infants	
	1 or both parents in study (n = 420)	Both parents in study (n = 228)
Male, No. (%)	217 (52)	116 (51)
Birth weight, g		
Mean (SD)	3363 (551)	3353 (516)
Range	1090-5040	1750-5040
Low birth weight (<2500 g), No. (%)	20 (5)	8 (4)
Gestational age at birth, wk		
Mean (SD)	39.3 (1.7)	39.3 (1.5)
Range	29-42	33-42
Preterm birth, No. (%)		
<37 wk	34 (8)	18 (8)
<32 wk	4 (1)	0
Mode of conception, No. (%)		
Assisted reproductive technology^a^	240 (57)	138 (61)
Non–assisted reproductive technology^b^	180 (43)	90 (39)

^a^ Fresh or frozen in vitro fertilization protocols, including intracytoplasmic sperm injection.

^b^ Intrauterine insemination with or without ovulation induction or stimulation, ovulation induction or stimulation with timed intercourse, or nonmedically assisted or naturally conceived.

### Urinary Biomarker Concentrations

In total, 1700 maternal preconception urine samples and 590 paternal preconception urine samples were analyzed for phthalate and DINCH metabolites. Women provided a mean (SD) of 4.1 (3.0) urine samles (median, 3 urine samples; interquartile range, 2-5 urine samples), and men provided a mean (SD) of 2.6 (1.7) urine samples (median, 2 urine samples; interquartile range, 1-3 urine samples). The distribution of metabolites, detection frequencies, and correlations can be found in eTable 1 and eTable 2 in the Supplement.

### Maternal Preconception Window

After adjusting for covariates, we found that maternal preconception urinary ΣDEHP metabolite concentrations were associated with increased risk of preterm birth (RR, 1.50; 95% CI, 1.09-2.06; *P* = .01). Risk ratios increased slightly in models accounting for maternal prenatal ΣDEHP concentrations (RR, 1.69; 95% CI, 1.17-2.44; *P* = .006) (Table 3). This association appeared to be stronger for male infants (RR, 2.01; 95% CI, 1.17-3.45) compared with female infants (RR, 1.22; 95% CI, 0.79-1.88) (effect-measure modification *P* = .17) (eTable 3 in the Supplement). Quartile analysis showed a positive dose-response association between maternal preconception urinary DEHP concentrations and preterm birth (eTable 4 in the Supplement). There was some suggestion that mothers with higher preconception urinary MHiNCH concentrations had an increased risk of preterm birth in the main covariate-adjusted model (RR, 1.70; 95% CI, 0.89-3.24; *P* = .11) (Table 3). This association was attenuated by additional adjustment for prenatal MHiNCH concentrations (RR, 1.17; 95% CI, 0.49-2.81*; P = *.72). In sensitivity analyses, maternal preconception MHiNCH concentrations above the median were associated with a suggested increased risk of preterm birth (RR, 4.02; 95% CI, 0.84-19.30; *P* = .08). Maternal preconception MHiNCH concentrations were associated with reduced gestational age (β = −2.01 days; 95% CI, −3.74 to −0.29 days; *P* = .02) (eTable 6 in the Supplement). No other maternal preconception metabolites were associated with the risk of preterm birth (Table 3).

**Table 3.** Data on Preterm Birth (<37 Weeks) per Natural Log-Unit Increase in Maternal Preconception Urinary Phthalate and DINCH Biomarker Concentrations Among 419 Mothers in the Environment and Reproductive Health Study, 2005-2018

Biomarker	Model 1 (unadjusted)			Model 2 (covariates)^a^			Model 3 (covariates plus prenatal)^b^			Model 4 (covariates plus paternal preconception)^c^		
	No./total No.	RR (95% CI)	*P* value	No./total No.	RR (95% CI)	*P* value	No./total No.	RR (95% CI)	*P* value	No./total No.	RR (95% CI)	*P* value
ΣDEHP^d^	34/419	1.49 (1.08-2.04)	.01	34/419	1.50 (1.09-2.06)	.01	31/386	1.69 (1.17-2.44)	.006	18/228	2.37 (1.39-3.70)	.001
MEHP	34/419	1.45 (1.03-2.04)	.03	34/419	1.51 (1.08-2.13)	.02	31/386	1.64 (1.13-2.37)	.009	18/228	2.38 (1.44-3.94)	<.001
MEHHP	34/419	1.44 (1.08-1.93)	.01	34/419	1.45 (1.08-1.95)	.01	31/386	1.59 (1.12-2.25)	.009	18/228	2.09 (1.34-3.26)	.001
MEOHP	34/419	1.47 (1.09-1.97)	.01	34/419	1.48 (1.10-2.00)	.01	31/386	1.59 (1.11-2.27)	.01	18/228	2.12 (1.36-3.31)	.001
MECPP	34/419	1.47 (1.06-2.04)	.02	34/419	1.49 (1.07-2.07)	.02	31/386	1.74 (1.17-2.59)	.006	18/228	2.30 (1.35-3.92)	.002
MBP	34/419	1.15 (0.79-1.67)	.47	34/419	1.16 (0.79-1.70)	.46	31/386	1.24 (0.74-2.07)	.41	18/228	1.25 (0.71-2.20)	.38
MiBP	34/419	0.76 (0.52-1.10)	.15	34/419	0.77 (0.53-1.14)	.19	31/386	0.83 (0.52-1.33)	.45	18/228	0.78 (0.44-1.38)	.39
MBzP	34/419	1.08 (0.77-1.50)	.65	34/419	1.13 (0.80-1.58)	.50	31/386	1.17 (0.73-1.87)	.51	18/228	0.95 (0.53-1.71)	.88
ΣAAPhthalates^e^	34/419	1.49 (1.07-2.07)	.02	34/419	1.51 (1.08-2.11)	.02	31/386	1.79 (1.19-2.72)	.006	18/228	2.97 (1.22-3.18)	.006
MCPP	34/419	0.88 (0.62-1.25)	.48	34/419	0.88 (0.62-1.25)	.49	31/386	0.84 (0.56-1.27)	.40	18/228	1.07 (0.60-1.92)	.81
MCOP	31/400	0.81 (0.61-1.09)	.16	31/400	0.83 (0.63-1.11)	.21	26/358	0.84 (0.58-1.23)	.38	15/212	0.93 (0.53-1.61)	.79
MCNP	31/400	1.06 (0.71-1.56)	.79	31/400	1.07 (0.73-1.59)	.71	26/358	1.19 (0.73-1.94)	.48	15/212	1.32 (0.66-2.67)	.44
MEP	34/419	1.04 (0.79-1.37)	.75	34/419	1.04 (0.79-1.37)	.77	31/386	0.99 (0.68-1.44)	.96	18/228	0.98 (0.64-1.49)	.93
MHiNCH	10/205	1.63 (0.91 2.94)	.10	10/205	1.70 (0.89-3.24)	.11	8/186	1.17 (0.49-2.81)	.72	2/97	3.96 (0.23-69.16)	.35
MCOCH	9/166	1.24 (0.51-2.99)	.63	9/166	1.17 (0.44-3.07)	.76	7/148	0.56 (0.15-2.15)	.40	2/75	4.10 (0.11-147.94)	.44

Abbreviations: DEHP, di(2-ethylhexyl) phthalate; DINCH, di(isononyl)cyclohexane-1,2-dicarboxylate; MBP, mono-n-butyl phthalate; MBzP, monobenzyl phthalate; MCNP, monocarboxyisononyl phthalate; MCOCH, cyclohexane-1,2-dicarboxylic acid monocarboxyisooctyl ester; MCOP, monocarboxyisooctyl phthalate; MCPP, mono(3-carboxypropyl) phthalate; MECPP, mono(2-ethyl-5-carboxypentyl) phthalate; MEHP, mono(2-ethylhexyl) phthalate; MEHHP, mono(2-ethyl-5-hydroxyhexyl) phthalate; MEOHP, mono(2-ethyl-5-oxohexyl) phthalate; MEP, monoethyl phthalate; MHiNCH, cyclohexane-1,2-dicarboxylic acid monohydroxy isononyl ester; MiBP, mono-isobutyl phthalate; RR, risk ratio.

^a^ Adjusted for age (continuous), body mass index (continuous), assisted reproductive technology (yes or no), smoking (ever or never), educational level (categorical).

^b^ Adjusted for age (continuous), body mass index (continuous), assisted reproductive technology (yes or no), smoking (ever or never), educational level (categorical) plus prenatal biomarker exposure (continuous log concentration).

^c^ Adjusted for age (continuous), body mass index (continuous), assisted reproductive technology (yes or no), smoking (ever or never), educational level (categorical) plus paternal preconception biomarker exposure (continuous log concentration).

^d^ Weighted molar sum of DEHP metabolites MEHP (molecular weight = 272), MEHHP (molecular weight = 294), MEOHP (molecular weight = 292), and MECPP (molecular weight = 308) concentrations expressed in units of micromoles per liter. We multiplied the molar sum by the molecular weight of MECPP (308 g/mol) to express ΣDEHP in units of nanograms per milliliter.

^e^ Calculated by multiplying the specific gravity–adjusted concentration of each of these 7 individual phthalate metabolites by their antiandrogenic potency and summing the weighted concentrations: ΣAAPhthalates = MBP + (0.24 × MiBP) + (0.26 × MBzP) + (0.61 × MEHP) + (0.61 × MEHHP) + (0.61 × MEOHP) + (0.61 × MECPP).

### Paternal Preconception Window

Paternal urinary ΣDEHP metabolite concentrations were associated with an increased risk of preterm birth in covariate-adjusted models (RR, 1.41; 95% CI, 0.94-2.11; *P* = .09) (Table 4). However, this association was markedly attenuated toward the null in models accounting for maternal preconception ΣDEHP concentrations (RR, 1.06; 95% CI, 0.66-1.68; *P* = .82). The results of MHiNCH and MCOCH models in association with preterm birth were imprecise owing to low power. In sensitivity analyses, paternal preconception MHiNCH or MCOCH concentrations were not associated with continuous gestational age (eTable 6 in the Supplement). The remaining paternal preconception biomarkers showed little evidence of an association with preterm birth (Table 4).

**Table 4.** Data on Preterm Birth (<37 Weeks) per Natural Log-Unit Increase in Paternal Preconception Urinary Phthalate and DINCH Biomarker Concentrations Among 229 Fathers in the Environment and Reproductive Health Study, 2005-2018

Biomarker	Model 1 (unadjusted)			Model 2 (covariates)^a^		Model 3 (covariates plus prenatal)^b^				Model 4 (covariates plus maternal preconception)^c^		
	No./total No.	RR (95% CI)	*P* value	No./total No.	RR (95% CI)	*P* value	No./total No.	RR (95% CI)	*P* value	No./total No.	RR (95% CI)	*P* value
ΣDEHP^d^	18/229	1.39 (0.98-1.98)	.07	18/229	1.41 (0.94-2.11)	.09	17/213	1.46 (0.88-2.43)	.14	18/228	1.06 (0.66-1.68)	.82
MEHP	18/229	1.34 (0.97-1.85)	.08	18/229	1.34 (0.92-1.94)	.13	17/213	1.34 (0.89-2.01)	.16	18/228	1.10 (0.73-1.67)	.63
MEHHP	18/229	1.39 (0.99-1.95)	.06	18/229	1.40 (0.96-2.06)	.08	17/213	1.38 (0.86-2.21)	.18	18/228	1.10 (0.71-1.69)	.68
MEOHP	18/229	1.37 (0.97-1.94)	.07	18/229	1.38 (0.93-2.06)	.11	17/213	1.37 (0.84-2.24)	.21	18/228	1.08 (0.68-1.69)	.75
MECPP	18/229	1.39 (0.98-1.98)	.07	18/229	1.41 (0.95-2.11)	.09	17/213	1.55 (0.93-2.60)	.10	18/228	1.04 (0.65-1.65)	.87
MBP	18/229	1.10 (0.66-1.82)	.72	18/229	1.06 (0.64-1.75)	.83	17/213	1.16 (0.64-2.11)	.62	18/228	0.97 (0.57-1.68)	.93
MiBP	18/229	0.66 (0.38-1.15)	.14	18/229	0.66 (0.38-1.15)	.15	17/213	0.79 (0.42-1.48)	.46	18/228	0.74 (0.40-1.38)	.35
MBzP	18/229	0.94 (0.58-1.53)	.80	18/229	0.93 (0.56-1.54)	.78	17/213	1.00 (0.57-1.77)	.97	18/228	0.96 (0.55-1.70)	.90
ΣAAPhthalates^e^	18/229	1.39 (0.94-2.04)	.10	18/229	1.38 (0.91-2.10)	.13	17/213	1.48 (0.88-2.47)	.14	18/228	1.13 (0.72-1.79)	.60
MCPP	18/229	0.70 (0.44-1.12)	.14	18/229	0.67 (0.41-1.09)	.10	17/213	0.72 (0.42-1.22)	.22	18/228	0.66 (0.39-1.10)	.11
MCOP	15/219	0.76 (0.51-1.14)	.18	15/219	0.76 (0.49-1.16)	.20	13/199	0.71 (0.40-1.23)	.22	15/212	0.76 (0.46-1.26)	.28
MCNP	15/219	0.89 (0.51-1.55)	.69	15/219	0.87 (0.49-1.55)	.64	13/199	0.96 (0.52-1.78)	.90	15/212	0.76 (0.40-1.45)	.40
MEP	18/229	1.00 (0.70-1.44)	.98	18/229	0.94 (0.64-1.39)	.76	17/213	0.99 (0.66-1.47)	.96	18/228	0.95 (0.63-1.43)	.81
MHiNCH	2/99	1.20 (0.26-5.55)	.82	2/99	DNC	NA	1/90	NA	DNC	2/97	DNC	NA
MCOCH	2/78	1.72 (0.32-9.26)	.53	2/78	DNC	NA	1/69	NA	DNC	2/75	DNC	NA

Abbreviations: DEHP, di(2-ethylhexyl) phthalate; DINCH, di(isononyl)cyclohexane-1,2-dicarboxylate; DNC, do not converge; MBP, mono-n-butyl phthalate; MBzP, monobenzyl phthalate; MCNP, monocarboxyisononyl phthalate; MCOCH, cyclohexane-1,2-dicarboxylic acid monocarboxyisooctyl ester; MCOP, monocarboxyisooctyl phthalate; MCPP, mono(3-carboxypropyl) phthalate; MECPP, mono(2-ethyl-5-carboxypentyl) phthalate; MEHP, mono(2-ethylhexyl) phthalate; MEHHP, mono(2-ethyl-5-hydroxyhexyl) phthalate; MEOHP, mono(2-ethyl-5-oxohexyl) phthalate; MEP, monoethyl phthalate; MHiNCH, cyclohexane-1,2-dicarboxylic acid monohydroxy isononyl ester; MiBP: mono-isobutyl phthalate; NA: not applicable; RR, risk ratio.

^a^ Adjusted for age (continuous), body mass index (continuous), assisted reproductive technology (yes or no), smoking (ever or never), educational level (categorical).

^b^ Adjusted for age (continuous), body mass index (continuous), assisted reproductive technology (yes or no), smoking (ever or never), educational level (categorical) plus prenatal biomarker exposure (continuous log concentration).

^c^ Adjusted for age (continuous), body mass index (continuous), assisted reproductive technology (yes or no), smoking (ever or never), educational level (categorical) plus paternal preconception biomarker exposure (continuous log concentration).

^d^ Weighted molar sum of DEHP metabolites MEHP (molecular weight = 272), MEHHP (molecular weight = 294), MEOHP (molecular weight = 292), and MECPP (molecular weight = 308) concentrations expressed in units of micromoles per liter. We multiplied the molar sum by the molecular weight of MECPP (308 g/mol) to express ΣDEHP in units of nanograms per milliliter.

^e^ Calculated by multiplying the specific gravity–adjusted concentration of each of these 7 individual phthalate metabolites by their antiandrogenic potency and summing the weighted concentrations: ΣAAPhthalates = MBP + (0.24 × MiBP) + (0.26 × MBzP) + (0.61 × MEHP) + (0.61 × MEHHP) + (0.61 × MEOHP) + (0.61 × MECPP).

### Couple-Based Sensitivity Analyses

In analyses restricted to 228 couples, associations of maternal preconception ΣDEHP metabolite concentrations with preterm birth remained robust in covariate-adjusted models (RR, 2.30; 95% CI, 1.46-3.60; *P* < .001), as well as in models additionally adjusting for prenatal (RR, 4.98; 95% CI, 2.31-10.75; *P* < .001) or paternal preconception (RR, 2.37; 95% CI, 1.39-3.70; *P* = .001) ΣDEHP concentrations (eTable 5 in the Supplement). Maternal preconception MHiNCH concentrations were associated with an increased risk of preterm birth among couples in an unadjusted model (RR, 3.48; 95% CI, 0.91-13.36; *P* = .07); however, associations became imprecise in models adjusting for covariates (RR, 3.15; 95% CI, 0.23-43.95; *P* = .39) (eTable 5 in the Supplement).

## Discussion

In this prospective cohort of subfertile couples, urinary ΣDEHP metabolite concentrations measured in mothers before conception were associated with a higher risk of singleton preterm birth. The results of ΣDEHP models were robust to adjustments for prenatal exposure. This association was more pronounced among male infants than female infants. Couple-based analyses confirmed the results for an association between maternal preconception ΣDEHP concentrations and increased risk of preterm birth. Maternal preconception MHiNCH concentrations were suggestively associated with an elevated risk of preterm birth. These results were further confirmed in a sensitivity analysis examining gestational age continuously. We found that a log-unit increase in maternal MHiNCH concentrations was associated with a reduction in gestational age by approximately 2 days. However, this association was partially explained by prenatal MHiNCH concentrations and should be interpreted cautiously owing to the small numbers and low detection frequencies. Future studies should confirm or rule out a potential association with this emerging phthalate substitute. We observed little evidence of associations between paternal preconception phthalate metabolites or biomarkers of plasticizer substitutes and preterm birth.

To our knowledge, this is the first study evaluating couples’ exposure to phthalate metabolites during the preconception window and its association with preterm birth. Previous human studies of preterm birth have assessed phthalate exposure during the prenatal window. Our maternal preconception ΣDEHP findings are compatible with most prior research,^14,16,17,47,48^ although not all,^49,50^ on prenatal phthalate exposure. Ferguson and colleagues^14,17^ used a nested case-control design and reported robust dose-response associations of ΣDEHP and MBP with increased odds of overall preterm birth and spontaneous preterm birth among North American women. A case-control study by Meeker et al^16^ also found a significantly increased risk of preterm birth in association with urinary concentrations of ΣDEHP and MBP among Mexican women. Gao et al^47^ reported positive associations between prenatal DEHP metabolites in Chinese mothers and preterm birth. In contrast, Ferguson et al^50^ found no association between prenatal DEHP metabolites and preterm birth in a recent analysis among Puerto Rican mothers, whereas Adibi et al^49^ reported inverse associations between prenatal DEHP metabolites and preterm birth among North American mothers. In a recent systematic review of the literature, Radke et al^18^ concluded that exposure to DEHP was associated with the risk of preterm birth, with a moderate level of evidence. In addition, studies have evaluated prenatal phthalates in association with continuous gestational age with heterogeneous epidemiologic methods; some reported shorter gestations,^51,52,53,54^ while others found inconsistent^55,56^ or contrasting^49,57^ results.

Our findings support a novel hypothesis: maternal phthalate exposure during the critical period before conception may be associated with shorter gestation. Although, to our knowledge, mechanistic data on preconception exposures are scarce, this latent association could be compatible with the established association of DEHP with the ovary and its related epigenetic modifications in oocytes.^22,23,58,59^ In addition, DEHP metabolites may disrupt nuclear receptors, including peroxisome proliferator–activated receptors, the androgen receptor, and estrogenic receptors,^25,60,61,62^ and they may increase oxidative stress and inflammation in the ovary and endometrium.^63,64,65^ It is accepted that early disruption to peri-implantation processes such as alterations in embryo spacing or development, decidualization, and placentation may perpetuate throughout pregnancy, manifesting later as preterm birth, among other adverse outcomes.^66^ Based on previous results from the EARTH Study team and those of others, including fertile populations, we hypothesize that an early action of DEHP metabolites at the ovary may interfere with normal fertility and implantation processes,^67,68^ predisposing to a syndrome of complications throughout gestation that may be associated with altered placental function,^69,70^ embryo and fetal growth restriction,^71^ preeclampsia,^72^ pregnancy loss,^73^ and ultimately preterm birth. Of relevance for preventive care, this syndrome could have its roots in the preconception or periconception period.^74^

### Strengths and Limitations

This study has some strengths. A major strength was the opportunity to assess maternal and paternal phthalate exposure before conception. Although the generalizability of our findings to fertile couples is uncertain, our results are consistent with those of previous studies reporting phthalate-associated adverse pregnancy outcomes in both subfertile and fertile populations, including a preconception cohort of fertile couples in which maternal preconception phthalate exposure was associated with reductions in gestational age and birth weight.^75^ In addition, the ongoing follow-up of the EARTH Study allowed for a timely assessment of the phthalate substitute DINCH; however, continued follow-up will allow us to strengthen this evidence. Another strength was the use of multiple urine samples, resulting in a more precise exposure assessment while reducing exposure misclassification and its expected attenuation bias.^76^ However, some degree of misclassification cannot be ruled out given the short biological half-lives and episodic nature of exposure to these nonpersistent chemicals. There has been recent interest in examining the association between the modification of environmental chemicals and preterm birth by levels of stress in pregnancy.^77^ Unfortunately, owing to the absence of any stress assessment in the EARTH Study, we could neither adjust for nor examine this association.

This study also has some limitations. One limitation was the modest number of preterm birth cases, which precluded us from studying clinical subtypes of preterm birth. Given that previous research has shown elevated odds of spontaneous preterm birth in association with prenatal phthalate exposure,^14^ future work with more cases should allow for the examination of subtypes of preterm birth. We also had limited power to detect sex-specific differences; these results should be interpreted with caution. We acknowledge that multiple comparisons were performed, and thus we cannot rule out that some of the associations could be due to chance. However, this possibility appears unlikely given the consistency of our results for the maternal preconception window showing positive associations with specific metabolites of DEHP (or phthalate substitutes), the absence of any associations within the paternal preconception window across all of the metabolites examined, and the attenuation of any potential paternal finding after accounting for maternal preconception biomarker exposure. Furthermore, the maternal preconception DEHP associations remained robust and internally consistent across all of the models analyzed. Last, our significant results have biological underpinnings from prior toxicologic studies and are consistent with previous epidemiologic evidence.

## Conclusions

In this prospective study, maternal preconception urinary ΣDEHP metabolite concentrations were associated with an increased risk of preterm birth. Our results suggest that female exposure to DEHP before conception might be an unrecognized risk factor for adverse pregnancy outcomes, often overlooked in clinical practice. These findings may have important clinical and public health implications, given the ubiquity of DEHP exposure, the importance of the outcome, and that prevention strategies rarely focus on preconception care. Although future studies should validate these associations, it is appropriate to inform couples planning conception about measures to reduce phthalate exposure.

## Notes

Supplement. **eTable 1.** Distribution of Specific Gravity–Normalized Geometric Mean Urinary Phthalate and DINCH Biomarker Concentrations From 419 Mothers and 229 Fathers in the Environment and Reproductive Health (EARTH) Study, 2005-2018 **eTable 2.** Spearman Correlation Coefficients for Paternal and Maternal Natural Log ΣDEHP Metabolite Concentrations **eTable 3.** Risk Ratios (95% CIs) for Preterm Birth (<37 weeks) per Log-Unit Increase in Maternal and Paternal Preconception Urinary Phthalate and DINCH Biomarker Concentrations by Infant Sex **eTable 4.** Risk Ratios (RR) for Preterm Birth (<37 weeks) Across Quartiles of Maternal Preconception Urinary di(2-ethylhexyl) Phthalate (DEHP) Concentrations in the Environment and Reproductive Health (EARTH) Study, 2005-2018 **eTable 5.** Risk Ratios (RR) for Preterm Birth (<37 weeks) per Log-Unit Increase in Maternal Preconception Urinary Phthalate and DINCH Biomarker Concentrations Among 228 Couples in the Environment and Reproductive Health (EARTH) Study, 2005-2018 **eTable 6.** Association of Natural Log-Unit Increase in Parental Preconception Urinary DINCH Metabolites Concentrations and Gestational Age (Days) **eFigure.** Hypothesized Directed Acyclic Graph (DAG) Between Maternal Preconception Phthalates and DINCH Exposure and Preterm Birth Risk
