# Supplementary material for: Association of Parental Preconception Exposure to Phthalates and Phthalate Substitutes With Preterm Birth
Source: JAMA Netw Open. 2020 Apr 7;3(4):e202159. doi: 10.1001/jamanetworkopen.2020.2159 (PMC7139277; doi:10.1001/jamanetworkopen.2020.2159)
Supplement: Supplement. — eTable 1. Distribution of Specific Gravity–Normalized Geometric Mean Urinary Phthalate and DINCH Biomarker Concentrations From 419 Mothers and 229 Fathers in the Environment and Reproductive Health (EARTH) Study, 2005-2018 eTable 2. Spearman Correlation Coefficients for Paternal and Maternal Natural Log ΣDEHP Metabolite Concentrations eTable 3. Risk Ratios (95% CIs) for Preterm Birth (<37 weeks) per Log-Unit Increase in Maternal and Paternal Preconception Urinary Phthalate and DINCH Biomarker Concentrations by Infant Sex eTable 4. Risk Ratios (RR) for Preterm Birth (<37 weeks) Across Quartiles of Maternal Preconception Urinary di(2-ethylhexyl) Phthalate (DEHP) Concentrations in the Environment and Reproductive Health (EARTH) Study, 2005-2018 eTable 5. Risk Ratios (RR) for Preterm Birth (<37 weeks) per Log-Unit Increase in Maternal Preconception Urinary Phthalate and DINCH Biomarker Concentrations Among 228 Couples in the Environment and Reproductive Health (EARTH) Study, 2005-2018 eTable 6. Association of Natural Log-Unit Increase in Parental Preconception Urinary DINCH Metabolites Concentrations and Gestational Age (Days) eFigure. Hypothesized Directed Acyclic Graph (DAG) Between Maternal Preconception Phthalates and DINCH Exposure and Preterm Birth Risk [file jamanetwopen-3-e202159-s001.pdf]

Supplementary Online Content

Zhang Y, Mustieles V, Yland J, et al. Association of parental preconception exposure to phthalates and phthalate substitutes with preterm birth. *JAMA Netw Open.* 2020;3(4):e202159. doi:10.1001/jamanetworkopen.2020.2159

eTable 1. Distribution of Specific Gravity–Normalized Geometric Mean Urinary Phthalate and DINCH Biomarker Concentrations From 419 Mothers and 229 Fathers in the Environment and Reproductive Health (EARTH) Study, 2005-2018
eTable 2. Spearman Correlation Coefficients for Paternal and Maternal Natural Log ΣDEHP Metabolite Concentrations
eTable 3. Risk Ratios (95% CIs) for Preterm Birth (<37 weeks) per Log-Unit Increase in Maternal and Paternal Preconception Urinary Phthalate and DINCH Biomarker Concentrations by Infant Sex
eTable 4. Risk Ratios (RR) for Preterm Birth (<37 weeks) Across Quartiles of Maternal Preconception Urinary di(2-ethylhexyl) Phthalate (DEHP) Concentrations in the Environment and Reproductive Health (EARTH) Study, 2005-2018
eTable 5. Risk Ratios (RR) for Preterm Birth (<37 weeks) per Log-Unit Increase in Maternal Preconception Urinary Phthalate and DINCH Biomarker Concentrations Among 228 Couples in the Environment and Reproductive Health (EARTH) Study, 2005-2018
eTable 6. Association of Natural Log-Unit Increase in Parental Preconception Urinary DINCH Metabolites Concentrations and Gestational Age (days)
eFigure. Hypothesized Directed Acyclic Graph (DAG) Between Maternal Preconception Phthalates and DINCH Exposure and Preterm Birth Risk

This supplementary material has been provided by the authors to give readers additional information about their work.

© 2020 Zhang Y et al. *JAMA Network Open.*

eTable 1. Distribution of Specific Gravity–Normalized Geometric Mean Urinary Phthalate and DINCH Biomarker Concentrations From 419 Mothers and 229 Fathers in the Environment and Reproductive Health (EARTH) Study, 2005-2018

Windows/ Biomarker	LOD (ng/ml)	Urine N [a]	% Detect [b]	SG-Adjusted GM (GSD) [c]	SG-Adjusted Median (ng/ml)	IQR 25th - 75th (ng/ml)
**Maternal Preconception**						
∑DEHP [d]	NA	1700	NA	42.8 (1.9)	36.5	22.1-79.2
MEHP	0.5-1.2	1700	69.76	2.2 (0.10)	2	1.2-4.0
MEHHP	0.2-0.7	1700	98.53	11.1 (0.55)	10.3	5.5-21.8
MEOHP	0.2-0.7	1700	97.71	7.2 (0.35)	6.7	3.6-14.2
MECPP	0.2-0.6	1700	91.35	19.3 (0.88)	17	10.1-35.4
MBP	0.4-0.6	1700	95.76	10.1 (0.42)	10.8	5.6-17.0
MiBP	0.2-0.3	1700	96.59	6.6 (0.28)	6.7	4.0-11.9
MBzP	0.2-0.3	1700	89.47	2.9 (0.14)	2.8	1.5-5.3
MCPP	0.1-0.2	1700	91.35	2.9 (0.14)	2.8	1.4-5.7
MCOP	0.2-0.7	1646	98.24	19.9 (1.2)	20.2	7.8-50.9
MCNP	0.2-0.6	1646	93.86	3.6 (0.16)	3.5	2.0-6.0
MEP	0.4-0.8	1700	99.65	49.1 (2.9)	44	20.8-92.7
MHiNCH	0.4	834	41.61	0.57(0.03)	0.5	<LOD-0.96
MCOCH	0.5	651	33.18	0.60(0.03)	0.6	<LOD-0.9
**Windows/ Biomarker**	**LOD (ng/ml)**	**Urine N [a]**	**% Detect [b]**	**SG-Adjusted GM (GSD) [c]**	**SG-Adjusted Median (ng/ml)**	**IQR 25th - 75th (ng/ml)**
**Paternal Preconception**						
∑DEHP [d]	NA	590	NA	55.2 (4.4)	51.4	22.1-114.6
MEHP	0.5-1.2	590	70.85	2.8 (0.24)	2.5	1.2-6.0
MEHHP	0.2-0.7	590	98.64	15.1 (1.3)	13.7	5.9-31.8
MEOHP	0.2-0.7	590	96.78	8.8 (0.74)	8.8	4.0-18.8
MECPP	0.2-0.6	590	94.24	24.2 (2.0)	23.3	9.9-48.7

© 2020 Zhang Y et al. *JAMA Network Open.*

MBP	0.4-0.6	590	95.93	9.4 (0.56)	9.4	5.4-16.0
MiBP	0.2-0.3	590	94.41	6.6 (0.36)	6.5	4.2-11.5
MBzP	0.2-0.3	590	93.56	2.9 (0.18)	3	1.4-5.4
MCPP	0.1-0.2	590	94.24	3.4 (0.24)	3	1.7-6.8
MCOP	0.2-0.7	573	98.08	22.2 (1.9)	23.3	8.3-58.2
MCNP	0.2-0.6	573	95.29	3.9 (0.24)	3.7	2.1-6.2
MEP	0.4-0.8	590	100.00	45.2 (3.8)	42.6	17.6-100.4
MHiNCH	0.4	281	39.86	0.52(0.05)	<LOD	<LOD-1.0
MCOCH	0.5	218	29.36	0.53(0.05)	<LOD	<LOD-0.9

Abbreviations: LOD: Limit of Detection; N: sample sizes; SG: specific gravity; GM: geometric mean; GSD: geometric standard deviation; IQR: interquartile range; 25th percentile (25th); 75th percentile (75th); DINCH: di(isononyl)cyclohexane-1,2-dicarboxylate; DEHP: di(2-ethylhexyl) phthalate; MEHP: mono(2-ethylhexyl) phthalate; MEHHP: mono(2-ethyl-5-hydroxyhexyl) phthalate; MEOHP: mono(2-ethyl-5-oxohexyl) phthalate; MECPP: mono(2-ethyl-5-carboxypentyl) phthalate; MBP: mono-n-butyl phthalate; MiBP: mono-isobutyl phthalate; MBzP: monobenzyl phthalate; MCPP: mono(3-carboxypropyl) phthalate; MCOP: monocarboxyisooctyl phthalate; MCNP: monocarboxyisononyl phthalate; MEP: monoethyl phthalate; MHiNCH: cyclohexane-1,2-dicarboxylic acid monohydroxy isononyl ester; MCOCH: cyclohexane-1,2-dicarboxylic acid monocarboxyisooctyl ester; NA: Not Applicable.

[a] Number of urine samples with phthalate metabolite: 1700 maternal preconception and 590 paternal preconception urines samples from 419 mothers and 229 fathers, respectively from the Environment and Reproductive Health (EARTH) study participants.
[b] Percentage of phthalate metabolite concentrations above the limit of detection (ng/ml). All values below the LOD (<LOD) were assigned a value equal to the LOD divided by √2.
[c] Geometric mean of urinary SG-adjusted concentrations expressed in ng/L.
[d] ∑DEHP, the weighted molar sum of metabolites MEHP (molecular weight=272), MEHHP (molecular weight=294), MEOHP (molecular weight=292) and MECPP (molecular weight=308) concentrations expressed in µmol/L. We multiplied the molar sum by the molecular weight of MECPP (308 g/mol) to express ∑DEHP as ng/ml.

© 2020 Zhang Y et al. *JAMA Network Open.*

eTable 2. Spearman Correlation Coefficients for Paternal and Maternal Natural Log ∑DEHP Metabolite Concentrations

Windows	Paternal Preconception	Maternal Preconception
Paternal Preconception	1	0.62
Maternal Preconception	0.62	1
Maternal Prenatal	0.59	0.60

© 2020 Zhang Y et al. *JAMA Network Open.*

eTable 3. Risk Ratios (95% CIs) for Preterm Birth (<37 weeks) per Log-Unit Increase in Maternal and Paternal Preconception Urinary Phthalate and DINCH Biomarker Concentrations by Infant Sex

	**Overall**		**Males**		**Females**		**Sex x Phthalate**
**Biomarker**	**RR (95% CI)**	**Preterm birth**	**RR (95% CI)**	**Preterm birth**	**RR (95% CI)**	**Preterm birth**	**EMM**
		**n / N**		**n / N**		**n / N**	***P* Value**
**Maternal Preconception**[a]							
∑DEHP [b]	1.50 (1.09, 2.06)	34/419	2.01 (1.17, 3.45)	16/216	1.22 (0.79, 1.88)	18/203	0.17
MEHP	1.51 (1.08, 2.13)	34/419	2.00 (1.15, 3.46)	16/216	1.12 (0.69, 1.83)	18/203	0.14
MBP	1.16 (0.79, 1.70)	34/419	1.05 (0.62, 1.77)	16/216	1.24 (0.68, 2.26)	18/203	0.81
MiBP	0.77 (0.53, 1.14)	34/419	0.76 (0.44, 1.33)	16/216	0.79 (0.46, 1.37)	18/203	0.96
MBzP	1.13 (0.80, 1.58)	34/419	1.07 (0.64, 1.78)	16/216	1.12 (0.70, 1.81)	18/203	0.94
∑AAPhth [c]	1.51 (1.08, 2.11)	34/419	1.71 (1.05, 2.78)	16/216	1.27 (0.79, 2.03)	18/203	0.32
MCPP	0.88 (0.62, 1.25)	34/419	0.92 (0.53, 1.58)	16/216	0.90 (0.58, 1.40)	18/203	0.99
MCOP	0.83 (0.63, 1.11)	31/400	0.90 (0.59, 1.36)	15/205	0.82 (0.55, 1.22)	16/195	0.63
MCNP	1.07 (0.73, 1.59)	31/400	1.11 (0.61, 1.99)	15/205	1.16 (0.67, 2.01)	16/195	0.80
MEP	1.04 (0.79, 1.37)	34/419	0.89 (0.57, 1.37)	16/216	1.18 (0.82, 1.70)	18/203	0.32
MHiNCH	1.70 (0.89, 3.24)	10/205	2.10 (0.78, 5.67)	5/102	1.98 (0.70, 5.63)	5/103	0.96
MCOCH	1.17 (0.44, 3.07)	9/166	1.24 (0.25, 6.13)	5/85	1.38 (0.27, 7.17)	4/81	0.99
**Paternal Preconception**[d]							
∑DEHP [b]	1.41 (0.94, 2.11)	18/229	1.25 (0.77, 2.04)	11/117	2.03 (0.94, 4.38)	7/112	0.30
MEHP	1.34 (0.92, 1.94)	18/229	1.12 (0.70, 1.79)	11/117	1.87 (0.95, 3.69)	7/112	0.20

© 2020 Zhang Y et al. *JAMA Network Open.*

	Overall		Males		Females		Sex x Phthalate
**Biomarker**	**RR (95% CI)**	**Preterm birth**	**RR (95% CI)**	**Preterm birth**	**RR (95% CI)**	**Preterm birth**	**EMM**
		**n / N**		**n / N**		**n / N**	***P* Value**
**Paternal Preconception[a]**							
MBP	1.06 (0.64, 1.75)	18/229	0.92 (0.48, 1.76)	11/117	1.45 (0.59, 3.59)	7/112	0.53
MiBP	0.66 (0.38, 1.15)	18/229	0.48 (0.21, 1.06)	11/117	0.92 (0.37, 2.28)	7/112	0.50
MBzP	0.93 (0.56, 1.54)	18/229	0.73 (0.38, 1.40)	11/117	1.41 (0.57, 3.48)	7/112	0.37
∑AAPhth [c]	1.38 (0.91, 2.10)	18/229	1.21 (0.72, 2.04)	11/117	1.99 (0.89, 4.43)	7/112	0.36
MCPP	0.67 (0.41, 1.09)	18/229	0.44 (0.20, 0.93)	11/117	1.10 (0.52, 2.33)	7/112	0.23
MCOP	0.76 (0.49, 1.16)	15/219	0.57 (0.28, 1.14)	9/110	1.08 (0.58, 2.02)	6/109	0.41
MCNP	0.87 (0.49, 1.55)	15/219	0.71 (0.32, 1.57)	9/110	1.60 (0.58, 4.43)	6/109	0.32
MEP	0.94 (0.64, 1.39)	18/229	0.94 (0.55, 1.60)	11/117	0.86 (0.44, 1.70)	7/112	0.95
MHiNCH	0.04 (0.01, 0.13)	2/99	2.98 (0.92, 9.70)	2/49	DNC	0/50	NA
MCOCH	0.02 (0.01, 0.07)	2/78	DNC	2/39	DNC	0/39	NA

Abbreviations: DINCH: di(isononyl)cyclohexane-1,2-dicarboxylate; DNC, Do Not Converge; DEHP: di(2-ethylhexyl) phthalate; MEHP: mono(2-ethylhexyl) phthalate; MEHHP: mono(2-ethyl-5-hydroxyhexyl) phthalate; MEOHP: mono(2-ethyl-5-oxohexyl) phthalate; MECPP: mono(2-ethyl-5-carboxypentyl) phthalate; MBP: mono-n-butyl phthalate; MiBP: mono-isobutyl phthalate; MBzP: monobenzyl phthalate; MCPP: mono(3-carboxypropyl) phthalate; MCOP: monocarboxyisooctyl phthalate; MCNP: monocarboxyisononyl phthalate; MEP: monoethyl phthalate; MHiNCH: cyclohexane-1,2-dicarboxylic acid monohydroxy isononyl ester; MCOCH: cyclohexane-1,2-dicarboxylic acid monocarboxyisooctyl ester; NA: Not Applicable.

[a]Adjusted for maternal age (continuous), BMI (continuous), ART (yes/no), smoking (ever/never), education (categorical).

[b]∑DEHP: is the weighted molar sum of DEHP metabolites MEHP (molecular weight=272), MEHHP (molecular weight=294), MEOHP (molecular weight=292) and MECPP (molecular weight=308) concentrations expressed in µmol/L. We multiplied the molar sum by the molecular weight of MECPP (308 g/mol) to express ∑DEHP as ng/ml.

[c]∑AAPhthalates: was calculated by multiplying the specific gravity adjusted concentration of each of these seven individual phthalate metabolites by their anti-androgenic potency and summing the weighted concentrations: ΣAAPhth = MBP + (0.24*MiBP) + (0.26*MBzP) + (0.61*MEHP) + (0.61*MEHHP) + (0.61*MEOHP) + (0.61*MECPP).

[d]Adjusted for maternal and paternal age (continuous), maternal and paternal BMI (continuous), ART (yes/no), maternal and paternal smoking (ever/never), education (categorical).

© 2020 Zhang Y et al. *JAMA Network Open.*

eTable 4. Risk Ratios (RR) for Preterm Birth (<37 weeks) Across Quartiles of Maternal Preconception Urinary di(2-ethylhexyl) Phthalate (DEHP) Concentrations in the Environment and Reproductive Health (EARTH) Study, 2005-2018

Maternal Preconception DEHP [a]	Preterm Birth		Preterm Birth		Preterm Birth	
(log-transformed)	Model2 [b]		Model3 [c]		Model4 [d]	
	Covariates		Covariates + Prenatal		Covariates + Paternal	
Quartile (range)	n/N	RR (95% CI)	n/N	RR (95% CI)	n/N	RR (95% CI)
q1 (1.21, 3.10)	7/104	ref	6/95	ref	1/57	ref
q2 (3.10, 3.60)	6/105	0.89 (0.30, 2.65)	5/97	0.85 (0.26, 2.81)	3/57	2.57 (0.26, 25.17)
q3 (3.60, 4.34)	7/105	1.03 (0.36, 2.97)	6/97	1.05 (0.32, 3.47)	3/57	2.81 (0.29, 27.36)
q4 (4.37, 8.03)	14/105	2.12 (0.85, 5.30)	14/97	2.76 (0.84, 9.01)	11/57	12.23 (1.55, 96.37)
p for trend		0.08		0.09		0.002

Abbreviations: n: Number of Preterm Birth; DEHP: di(2-ethylhexyl) phthalate; MEHP: mono(2-ethylhexyl) phthalate; ref: Reference Group.

[a] ∑DEHP: is the weighted molar sum of DEHP metabolites MEHP (molecular weight=272), MEHHP (molecular weight=294), MEOHP (molecular weight=292) and MECPP (molecular weight=308) concentrations expressed in µmol/L. We multiplied the molar sum by the molecular weight of MECPP (308 g/mol) to express ∑DEHP as ng/ml.

[b] Models 2: Adjusted for age (continuous), BMI (continuous), ART (yes/no), smoking (ever/never), education (categorical).

[c] Models 3: Adjusted for age (continuous), BMI (continuous), ART (yes/no), smoking (ever/never), education (categorical) + prenatal biomarker exposure (continuous log concentration).

[d] Models 4: Adjusted for age (continuous), BMI (continuous), ART (yes/no), smoking (ever/never), education (categorical) + paternal preconception biomarker exposure (continuous log concentration). [a] ∑DEHP: is the weighted molar sum of DEHP metabolites MEHP (molecular weight=272), MEHHP (molecular weight=294), MEOHP (molecular weight=292) and MECPP (molecular weight=308) concentrations expressed in µmol/L. We multiplied the molar sum by the molecular weight of MECPP (308 g/mol) to express ∑DEHP as ng/ml.

© 2020 Zhang Y et al. *JAMA Network Open.*

eTable 5. Risk Ratios (RR) for Preterm Birth (<37 weeks) per Log-Unit Increase in Maternal Preconception Urinary Phthalate and DINCH Biomarker Concentrations Among 228 Couples in the Environment and Reproductive Health (EARTH) Study, 2005-2018

	**Preterm Birth**			**Preterm Birth**			**Preterm Birth**			**Preterm Birth**		
**Biomarker**	**Model 1 [a]**			**Model 2 [b]**			**Model 3 [c]**			**Model 4 [d]**		
		**Unadjusted**		**Covariates**			**Covariates + Prenatal**			**Covariates + Paternal**		
	n/N	RR (95% CI)	*P* Values	n/N	RR (95% CI)	*P* Values	n/N	RR (95% CI)	*P* Values	n/N	RR (95% CI)	*P* Values
∑DEHP [e]	18/228	2.10 (1.39, 3.19)	<0.001	18/228	2.30 (1.46, 3.60)	<0.001	17/212	4.98 (2.31, 10.75)	<0.001	18/228	2.37 (1.39, 3.70)	0.001
MEHP	18/228	2.19 (1.43, 3.37)	<0.001	18/228	2.45 (1.51, 3.96)	<0.001	17/212	3.58 (1.87, 6.86)	<0.001	18/228	2.38 (1.44, 3.94)	<0.001
MEHHP	18/228	1.98 (1.35, 2.90)	<0.001	18/228	2.14 (1.42, 3.23)	<0.001	17/212	4.37 (2.09, 9.12)	<0.001	18/228	2.09 (1.34, 3.26)	0.001
MEOHP	18/228	2.00 (1.36, 2.94)	<0.001	18/228	2.16 (1.43, 3.26)	<0.001	17/212	4.22 (2.03, 8.76)	<0.001	18/228	2.12 (1.36, 3.31)	0.001
MECPP	18/228	2.13 (1.36, 3.32)	<0.001	18/228	2.32 (1.43, 3.75)	<0.001	17/212	4.82 (2.26, 10.27)	<0.001	18/228	2.30 (1.35, 3.92)	0.002
MBP	18/228	1.21 (0.74, 1.99)	0.44	18/228	1.25 (0.73, 2.12)	0.42	17/212	1.66 (0.73, 3.78)	0.23	18/228	1.25 (0.71, 2.20)	0.38
MiBP	18/228	0.71 (0.44, 1.15)	0.16	18/228	0.69 (0.42, 1.13)	0.14	17/212	0.84 (0.44, 1.61)	0.60	18/228	0.78 (0.44, 1.38)	0.39
MBzP	18/228	0.88 (0.54, 1.43)	0.60	18/228	0.95 (0.56, 1.59)	0.84	17/212	0.96 (0.49, 1.90)	0.92	18/228	0.95 (0.53, 1.71)	0.88
∑AAPhth [f]	18/228	1.85 (1.24, 2.77)	0.003	18/228	2.04 (1.30, 3.19)	0.002	17/212	4.36 (1.98, 9.62)	<0.001	18/228	2.97 (1.22, 3.18)	0.006

© 2020 Zhang Y et al. *JAMA Network Open.*

MCPP	18/228	0.88 (0.52, 1.48)	0.62	18/228	0.89 (0.51, 1.54)	0.67	17/212	0.96 (0.51, 1.83)	0.91	18/228	1.07 (0.60, 1.92)	0.81
MCOP	15/212	0.78 (0.50, 1.22)	0.27	15/212	0.79 (0.50, 1.25)	0.31	13/193	0.72 (0.39, 1.31)	0.28	15/212	0.93 (0.53, 1.61)	0.79
MCNP	15/212	1.13 (0.62, 2.06)	0.68	15/212	1.18 (0.62, 2.22)	0.62	13/193	1.39 (0.62, 3.14)	0.42	15/212	1.32 (0.66, 2.67)	0.44
	**Preterm Birth**			**Preterm Birth**			**Preterm Birth**			**Preterm Birth**		
**Biomarker**	**Model 1 [a]**			**Model 2 [b]**			**Model 3 [c]**			**Model 4 [d]**		
		**Unadjusted**		**Covariates**			**Covariates + Prenatal**			**Covariates + Paternal**		
	n/N	RR (95% CI)	*P* Values	n/N	RR (95% CI)	*P* Values	n/N	RR (95% CI)	*P* Values	n/N	RR (95% CI)	*P* Values
MEP	18/228	1.01 (0.68, 1.50)	0.97	18/228	0.97 (0.65, 1.45)	0.89	17/212	0.97 (0.57, 1.65)	0.91	18/228	0.98 (0.64, 1.49)	0.93
MHiNCH	2/97	3.48 (0.91, 13.36)	0.07	2/97	3.15 (0.23, 43.95)	0.39	1/88	DNC	NA	2/97	3.96 (0.23, 69.16)	0.35
MCOCH	2/75	6.82 (0.40, 115.64)	0.18	2/75	3.64 (0.11, 122.68)	0.47	1/66	DNC	NA	2/75	4.10 (0.11, 147.94)	0.44

Abbreviations: n: Number of Preterm Birth; DINCH: di(isononyl)cyclohexane-1,2-dicarboxylate; DEHP: di(2-ethylhexyl) phthalate; MEHP: mono(2-ethylhexyl) phthalate; MEHHP: mono(2-ethyl-5-hydroxyhexyl) phthalate; MEOHP: mono(2-ethyl-5-oxohexyl) phthalate; MECPP: mono(2-ethyl-5-carboxypentyl) phthalate; MBP: mono-n-butyl phthalate; MiBP: mono-isobutyl phthalate; MBzP: monobenzyl phthalate; MCPP: mono(3-carboxypropyl) phthalate; MCOP: monocarboxyisooctyl phthalate; MCNP: monocarboxyisononyl phthalate; MEP: monoethyl phthalate; MHiNCH: cyclohexane-1,2-dicarboxylic acid monohydroxy isononyl ester; MCOCH: cyclohexane-1,2-dicarboxylic acid monocarboxyisooctyl ester; DNC: Do Not Converge; NA: Not Applicable.

[a]Models 1: Unadjusted.

[b]Models 2: Adjusted for age (continuous), BMI (continuous), ART (yes/no), smoking (ever/never), education (categorical).

[c]Models 3: Adjusted for age (continuous), BMI (continuous), ART (yes/no), smoking (ever/never), education (categorical) + prenatal biomarker exposure (continuous log concentration).

[d]Models 4: Adjusted for age (continuous), BMI (continuous), ART (yes/no), smoking (ever/never), education (categorical) + paternal preconception biomarker exposure (continuous log concentration).

[e] ∑DEHP: is the weighted molar sum of DEHP metabolites MEHP (molecular weight=272), MEHHP (molecular weight=294), MEOHP (molecular weight=292) and MECPP (molecular weight=308) concentrations expressed in µmol/L. We multiplied the molar sum by the molecular weight of MECPP (308 g/mol) to express ∑DEHP as ng/ml.

[f] ∑AAPhthalates: was calculated by multiplying the specific gravity adjusted concentration of each of these seven individual phthalate metabolites by their anti-androgenic potency and summing the weighted concentrations: ΣAAPhth = MBP + (0.24*MiBP) + (0.26*MBzP) + (0.61*MEHP) + (0.61*MEHHP) + (0.61*MEOHP) + (0.61*MECPP).

© 2020 Zhang Y et al. *JAMA Network Open.*

eTable 6. Association of Natural Log-Unit Increase in Parental Preconception Urinary DINCH Metabolites Concentrations and Gestational Age (days)

**DINCH Metabolites**	**Maternal Preconception**[a]			**Paternal Preconception**[b]		
	Beta (95%CI)	*P* Value	N	Beta (95%CI)	*P* Value	N
MHiNCH	-2.01 (-3.74, -0.29)	0.02	205	0.18 (-1.88, 2.24)	0.86	99
MCOCH	-1.66 (-4.06, 0.75)	0.18	166	-0.73(-3.48, 2.02)	0.60	78

Abbreviations: DINCH: di(isononyl)cyclohexane-1,2-dicarboxylate; MHiNCH: cyclohexane-1,2-dicarboxylic acid monohydroxy isononyl ester; MCOCH: cyclohexane-1,2-dicarboxylic acid monocarboxyisooctyl ester.

[a]Adjusted for maternal age (continuous), BMI (continuous), ART (yes/no), smoking (ever/never), education (categorical).

[d]Adjusted for maternal and paternal age (continuous), maternal and paternal BMI (continuous), ART (yes/no), maternal and paternal smoking (ever/never), education (categorical).

© 2020 Zhang Y et al. *JAMA Network Open.*

eFigure. Hypothesized Directed Acyclic Graph (DAG) Between Maternal Preconception Phthalates and DINCH Exposure and Preterm Birth Risk

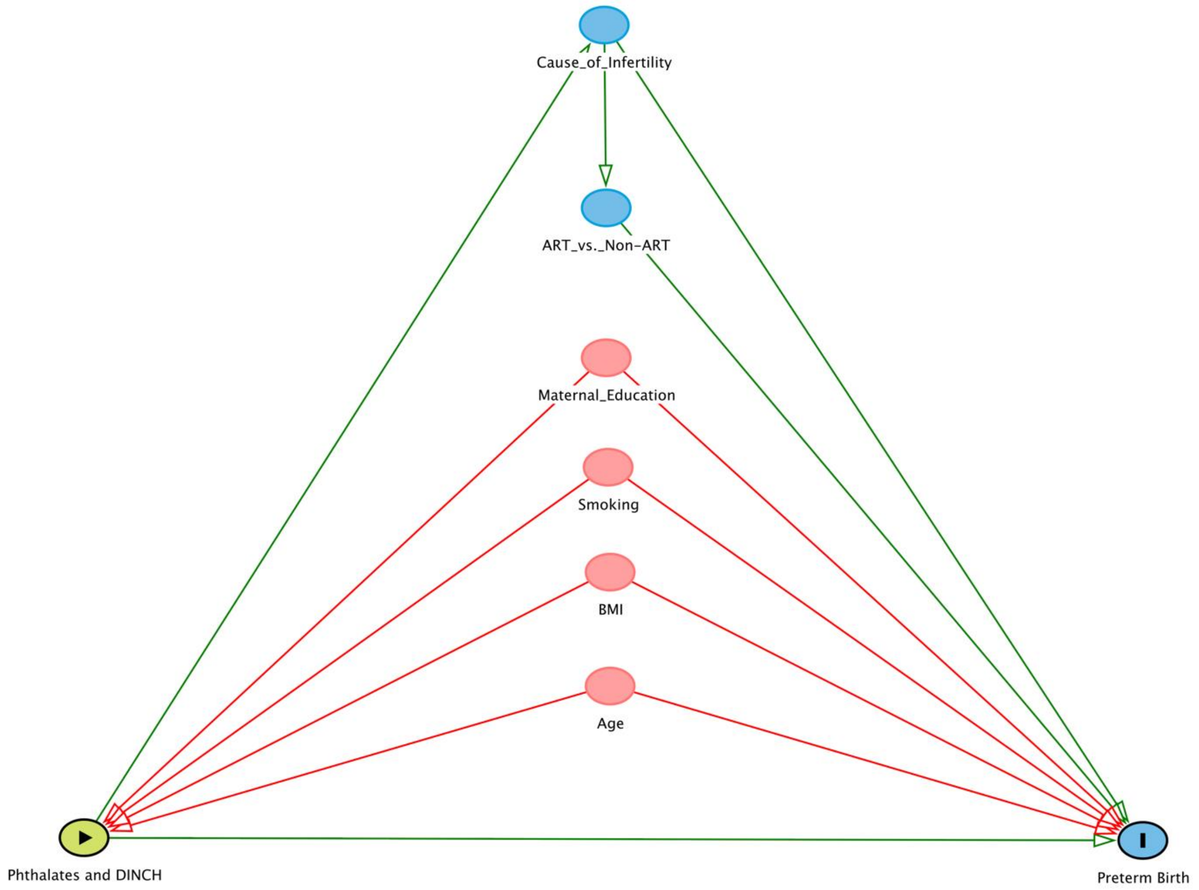

Notes: Potential confounders were selected *a priori* with a directed acyclic graph (DAG) using the Dagitty software. The DAG was drawn based on substantive knowledge of previous studies on phthalates and preterm birth. Blue circles are ancestors of the outcome and pink circles are ancestors of both exposure and outcome. Abbreviations: di(isononyl)cyclohexane-1,2-dicarboxylate (DINCH); body mass index (BMI); assisted reproductive technology (ART).

© 2020 Zhang Y et al. *JAMA Network Open.*
